# Apical Extrusion of Debris Produced during Continuous Rotating and Reciprocating Motion

**DOI:** 10.1155/2015/267264

**Published:** 2015-10-12

**Authors:** Giselle Nevares, Felipe Xavier, Luciana Gominho, Flávia Cavalcanti, Marcely Cassimiro, Kaline Romeiro, Pamella Alvares, Gabriela Queiroz, Ana Paula Sobral, Marleny Gerbi, Marcia Silveira, Diana Albuquerque

**Affiliations:** ^1^Department of Operative Dentistry and Endodontics, Dental College of Pernambuco, University of Pernambuco, Avenida Gal Newton Cavalcanti 1650, Tabatinga, 54753-901 Camaragibe, PE, Brazil; ^2^Odontologia, Unidade de Ciências Biológicas, UFCG, 58429-900 Campina Grande, PB, Brazil

## Abstract

This study aimed to analyse and compare apical extrusion of debris in canals instrumented with systems used in reciprocating and continuous motion. Sixty mandibular premolars were randomly divided into 3 groups (*n* = 20): the Reciproc (REC), WaveOne (WO), and HyFlex CM (HYF) groups. One Eppendorf tube per tooth was weighed in advance on an analytical balance. The root canals were instrumented according to the manufacturer's instructions, and standardised irrigation with 2.5% sodium hypochlorite was performed to a total volume of 9 mL. After instrumentation, the teeth were removed from the Eppendorf tubes and incubated at 37°C for 15 days to evaporate the liquid. The tubes were weighed again, and the difference between the initial and final weight was calculated to determine the weight of the debris. The data were statistically analysed using the Shapiro-Wilk, Wilcoxon, and Mann-Whitney tests (*α* = 5%). All systems resulted in the apical extrusion of debris. Reciproc produced significantly more debris than WaveOne (*p* < 0.05), and both systems produced a greater apical extrusion of debris than HyFlex CM (*p* < 0.001). Cross section and motion influenced the results, despite tip standardization.

## 1. Introduction

Apical debris extrusion may be clinically associated with pain and/or swelling in the presence of an intense inflammatory response [1]. This extrusion is an undesired consequence of the mechanical instrumentation of the root canal, and none of the available instrumentation systems can avoid apical debris extrusion. Thus, methods to minimize this phenomenon are continuously investigated [2]. Apical debris extrusion has been demonstrated to vary based on kinematics, number of files used, taper, cross section, and cutting efficacy [3], and these findings justify the need for an analysis of the widely used systems.

The Reciproc (VDW, Munich, Germany) and WaveOne (Dentsply/Maillefer, Ballaigues, Switzerland) single-file systems feature a specific motor that performs the reciprocating motion (i.e., movements alternating clockwise and counterclockwise) and are recommended for single use. Both systems are made of heat-treated nickel-titanium (*Memory Wire*, Dentsply Tulsa Dental Specialties, Tulsa, OK, USA), which is resistant to fatigue [4]. The reciprocating motion also improves the resistance of the nickel-titanium instrument to cyclical fatigue [5].

The HyFlex CM multiple-file system (Coltene Whaledent, Cuyahoga Falls, OH, USA) was developed for use in continuous rotation and is composed of a modified NiTi alloy (52 Ni wt% versus 54.5–57 Ni wt% in conventional NiTi alloys). This alloy undergoes Controlled Memory (CM) thermomechanical surface treatment, which increases the fatigue resistance by 150% and 390% compared with M-Wire and non-surface-treated conventional NiTi alloy, respectively [6]. Due to the lack of shape memory, this system enables visual functionality verification. The shape and strength of files with straightened spirals can be restored during autoclaving and reused, but files that do not return to their original shape should be discarded [7]. This property confers safety for up to three clinical uses [8].

This study compared the performance of the Reciproc, WaveOne, and HyFlex CM instruments based on the apical extrusion of debris produced during root canal preparation. To date, the apical debris extrusion resulting from the use of these three systems has not been compared. The null-hypothesis tested in this study stated that the amount of apically extruded debris does not differ between these instrumentation systems.

## 2. Methodology

### 2.1. Sample Selection

After review and approval by the University of Pernambuco (Pernambuco, Brazil) Research Ethics Committee, the samples were selected according to the following criteria: lower premolar with intact roots, complete root formation, and intact pulp chamber. The teeth were buccolingually and mesiodistally radiographed, and unique and straight canals were selected (<10°) [9]. Curvature angles were measured using the Image J program (US National Institutes of Health, Bethesda, MD, USA). The teeth were disinfected in a solution of 0.1% thymol for 24 h and stored in saline until the samples were used. Endodontic access was performed, and a glide path was created using a #10 file until the tip of the file could be observed in the apical foramen. Teeth in which the #20 file had adapted in the foramen were included, and those in which the #20 file became loose or did not reach were excluded. Procedures were executed with the help of a Dental Operating Microscope (DF Vasconcelos S/A, São Paulo, SP) at a magnification of 8x. The teeth had crowns that were worn with a carborundum disk until the teeth reached a total length of 17 mm, and the WL was set 1 mm short of the apical foramen. In total, 60 teeth met the inclusion criteria. After numbering the samples, the samples were randomized into three groups (*n* = 20) by a computer (https://www.random.org/).

### 2.2. Initial Weighing of the Eppendorf Tubes

**Figure 1:**
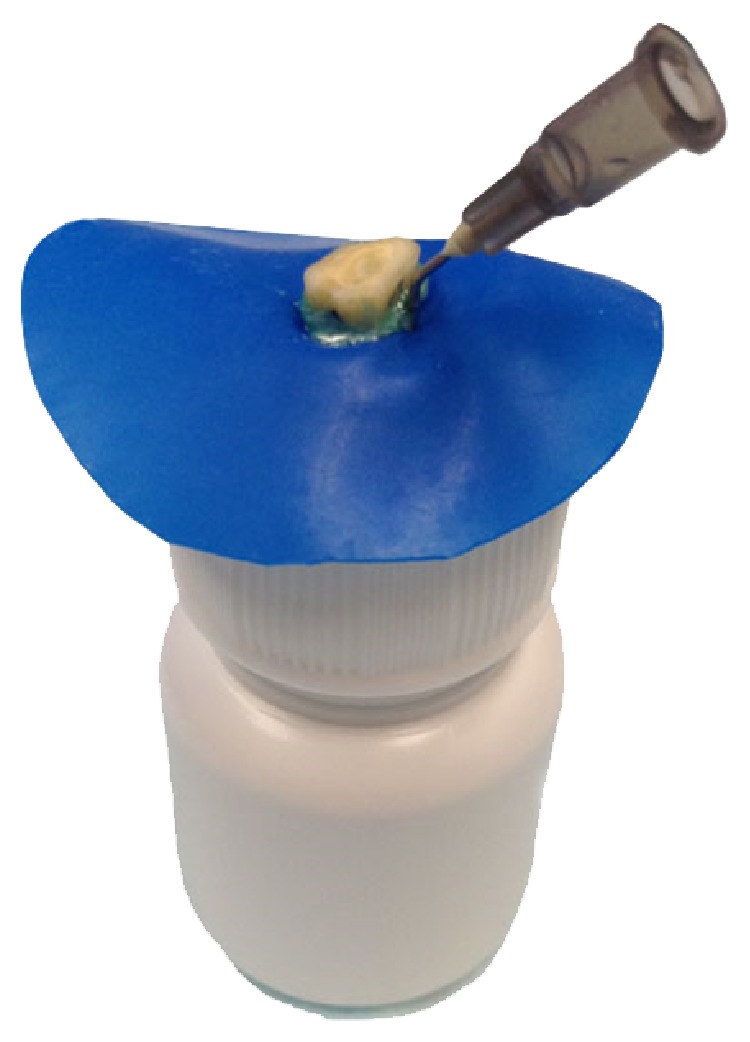
Modified apparatus used to evaluate the apical extrusion of debris.

The experimental model described by Myers and Montgomery (1991) [10] was used to evaluate the debris extrusion (Figure 1). An Eppendorf tube (Eppendorf AG, Hamburg, Germany) was numbered for each sample, and a hole was made in its lid. The Eppendorf tubes were individually weighed on an analytical balance with an accuracy of 0.0001 g (Denver Instrument GmbH XP series, Göttingen, Germany), and each tube was weighed five times. The heaviest and lightest weights were discarded, and the arithmetic mean of the remaining three weights was regarded as the starting weight of the Eppendorf tube. To prevent the accidental leakage of the irrigating solution during the experiment, the apparatus was covered with a rubber sheet after fixing the root in the tube lid with cyanoacrylate. The Eppendorf tube was placed in an opaque bottle to prevent the operator from being able to see the root canal during instrumentation. A 27G needle was folded and inserted in the Eppendorf lid to equalize the internal and external pressures.

### 2.3. Root Canal Preparation

The laboratory procedures were performed by a single operator. The groups were distributed according to the instrumentation system used. In all groups, the pulp chamber and canal were initially flooded with 2 mL of 2.5% sodium hypochlorite (NaOCl) before starting instrumentation.

#### 2.3.1. Reciproc (REC) Group

The Reciproc R40 (40/.06) file was coupled to a VDW Silver (VDW) motor under Reciproc programming. The file was used in a smooth back-and-forth motion with an amplitude of 3 mm. After three passes, the blades were cleaned with a dense sponge. Next, the canal was irrigated with 2 mL of 2.5% NaOCl. A #15 file was then used to maintain patency. These procedures were repeated three times until the file reached the WL. Irrigation was performed using a syringe and an open-end 30G needle (NaviTip, Ultradent Products, Utah, USA) in back-and-forth motion and positioned 2 mm short of the WL. The Reciproc file was discarded after use, and the teeth were removed from the Eppendorf tubes. The roots were washed with 1 mL NaOCl to collect the debris that had adhered to the outer surface. The total volume of irrigating solution was 9 mL per root.

#### 2.3.2. WaveOne (WO) Group

The WaveOne Large (40/.08) file was coupled to a VDW Silver motor using the WaveOne programming. Instrumentation and irrigation were performed in the same manner as in the REC group.

#### 2.3.3. HyFlex CM (HYF) Group

The single-length technique was used as recommended by the manufacturer [7] in the following sequence: 25/.08, 20/.04, 25/.04, 20/.06, 30/.04, and 40/.04. All files were attached to the VDW Silver motor that was set to a continuous rotation schedule at a speed of 500 rpm and a torque of 2.5 N-cm. The 25/.08 file was used in the cervical and middle thirds, whereas all other files reached the WL. After removal from the root canal, all files were cleaned with a dense sponge, the canal was irrigated with 1 mL of 2.5% NaOCl, and a #15 file was used to maintain patency. Each sorted kit was used three times and then discarded. The irrigation method, total volume of irrigating solution, and root washing procedure were the same as those described for the REC and WO groups.

### 2.4. Final Weighing of the Eppendorf Tubes

All tubes were incubated at 37°C in a biological incubator for 15 days to evaporate the remaining irrigation solution from the tubes. After the incubation period, the final weight was measured following the same method as that used to determine the initial weight. The Shapiro-Wilk statistical test was used to verify the normality of the data. Wilcoxon and pairwise Student's t-tests were used to verify the debris extrusion in the same group. To compare the results among groups, the data were analysed using the Kruskal-Wallis test. The equality of variance of sorted file diameters was calculated using the Levene test. As a result, the Tamhane test was employed to assess significant differences between groups. The margin of error used in the statistical tests was 5%.

## 3. Results

Apical debris extrusion was observed in all groups (Table 1). The intragroup analyses indicated significant differences between the initial and final weights in all groups (*p* < 0.05).

The intergroup analysis demonstrated that significantly more debris was extruded in the REC group than in the WO group (*p* < 0.05), and both of these groups exhibited more debris extrusion than the HYF group (*p* < 0.001).

## 4. Discussion

To the best of our knowledge, the apical debris extrusion associated with the use of the Reciproc, WaveOne, and HyFlex CM systems has not yet been compared. Apical debris extrusion occurred in all samples in this study, corroborating the results of previous studies in which all canal preparation protocols resulted in apical debris extrusion [11–13]. The tested null-hypothesis was rejected because the amount of apically extruded debris significantly differed among the groups (*p* < 0.05).

The number of instruments and the kinematics may contribute to debris extrusion during the instrumentation technique [2]. In this study, the multiple-file group exhibited less extrusion than the single-file groups, which is consistent with findings reported by Bürklein and Schäfer (2012) [14]. However, other studies observed more extrusion with the use of multiple-file systems compared with a single-file system [15], whereas the number of instruments used and the apical debris extrusion were not related [16]. Importantly, the single-file instrument was associated with reciprocating motion in all aforementioned studies. A comparison of the reciprocating single-file systems in the present study indicated that the extrusion associated with the Reciproc system exceeded that associated with the WaveOne system, and this difference corroborated previous findings [14]. Despite being used with the same motion, the Reciproc file is used with counterclockwise and clockwise rotation angles of 150° and 30°, respectively, at 300 rpm, whereas the respective angles used with the WaveOne file are 170° and 50° at 350 rpm [17]. However, the actual kinematic values for reciprocating instrumentation differ from the manufacturers' declared values and are more complex than descriptions of only angles and rotational speed [18]. Comparisons between single-file systems in continuous rotation (OneShape and F360) and reciprocating motion (Reciproc) showed less apical debris extrusion with continuous rotation systems [12]. Thus, more studies isolating the number of files used and kinematics are necessary to clarify these findings.

Regarding instrument design, the Reciproc system features a cross-sectional S-shape along the entire length of the working part and sharp cutting edges. The WaveOne system features a modified triangular cross section and the neutral rake angle that modifies to a convex triangular transverse cross section in the middle and neck portions of the working part of the instrument [12, 19]. Additionally, the HyFlex CM system features a slightly convex triangular cross section [20]. The results of this study may be due to the greater cutting efficiency attributed to the Reciproc system. During root canal instrumentation with the HyFlex CM system, the spirals unwound in 95% of the instruments (114/120 uses). Capar et al. (2014) [21] found that 80% of instruments were distorted. The lower extrusion rate in the HYF group could be related to this design modification, which could reduce the cutting efficiency and the amount of collected debris. Furthermore, unlike the Reciproc and HyFlex CM systems, the WaveOne file shows radial lands, and this feature can reduce the coronal debris removal capacity, enhancing apical debris extrusion [22].

The expansion of the apical diameter promoted by the instruments may also influence the amount of extruded debris. Specifically, the enlargement may directly correlate with the extent of extrusion [23]. Premolars with fully formed apices and straight canals were selected to minimize complications, such as the loss of WL or lack of a standardized preparation. A microcomputerized tomographic analysis of the canal morphology of premolars showed that the canal diameters 1 mm from the apex were >0.30 mm in the shorter diameter and >0.40 mm in the longer diameter [24]. A major goal of mechanical preparation is to touch all root canal walls, which would justify this methodology with an expansion at the WL of *D*_0_ 0.40 mm for both groups. Despite tip standardization, the taper differs among the studied systems, which may have influenced the extrusion outcome. The Reciproc R40 (.06 taper) and WaveOne Large (.08 taper) files feature a constant taper in the first 3 mm of the working part that decreases to *D*_16_ [19, 25], and the HyFlex CM system features a continuous .04 taper. The greater taper at the tip of the WaveOne and Reciproc files may promote greater debris extrusion compared with the HyFlex CM systems due to the greater preparation of the dentinal walls, which differs from the findings of the present study.

The methodology used in this study was based on a study by Myers and Montgomery (1991) [10, 12, 15, 26]. The apparatus used to collect debris was slightly modified from the previously proposed apparatus, in that the Eppendorf tube was supported in an opaque jar, which limited operator viewing during the procedures. Previous studies attempted to create a barrier using agarose gel [27, 28] and floral foam [29, 30]. However, the density of agarose gel does not simulate the same conditions as intact periapical tissue or tissue with periradicular lesions [28]. Furthermore, the sponge can absorb the irrigating solution and extruded debris, which hinders quantification [14]. To date, no method has been able to ideally simulate periapical tissue. Moreover, irrigation could be considered one of the primary causes of apical debris extrusion because instrumentation with irrigation produces extrusion, whereas instrumentation without irrigation does not produce any collectible debris [31]. In the current study, NaOCl was used to better simulate the clinical condition, and the use of this solution is well established [32]. However, the weight of the debris extruded using this method tended to exceed that obtained with a methodology using bidistilled water [12] because NaOCl crystallizes after extrusion. The volume of irrigating solution and the needle position were standardized, minimizing possible bias.

## 5. Conclusions

Under the experimental conditions of the present study, all systems caused apical debris extrusion. Reciprocating single-file systems were associated with higher debris extrusion than a multiple-file conventional rotary system.

## Figures and Tables

**Table 1:** Mean, maximum (max.) and minimum (min.) extrusion quantity (grams), standard deviation (SD), and number of teeth per group (*n*).

Groups	Mean	SD	Max.	Min.	*n*
REC	0.108	0.024	0.183	0.106	20
WO	0.097	0.004	0.104	0.089	20
HYF	0.080	0.007	0.085	0.083	20
